# Interstitial Lung Disease in Rare Congenital Syndromes

**DOI:** 10.34763/jmotherandchild.2020241.1931.000004

**Published:** 2020-07-29

**Authors:** Aleksandra Jezela-Stanek

**Affiliations:** 1 Department of Genetics and Clinical Immunology, National Institute of Tuberculosis and Lung Diseases, Warsaw, Poland

**Keywords:** interstitial lung disease, congenital malformation syndrome, genetics

## Abstract

Diffuse or interstitial lung disease (DLD/ILD) comprises a diverse group of disorders that involve the pulmonary parenchyma. Its aetiology varies (which makes the diagnostic process difficult), but congenital diseases, including malformation syndromes or developmental disorders, constitute one of the causative factors. They are rare conditions, and thus their frequency is not high. However, considering the progress and increasing availability of genetic testing, detection of these rare syndromes may increase. The aim of this work is, therefore, to present the symptomatology of selected congenital syndromes with ILD, taking into account the genetic background.

## Introduction

Diffuse lung disease (DLD), also known as interstitial lung disease (ILD) or diffuse parenchymal lung disease (DPLD) (1), comprises a diverse group of disorders that involve the pulmonary parenchyma. Some of them (in terms of causes) are similar in children and adults, but there are also certain diseases known to be unique to children (2, 3, 4, 5). Therefore, the term childhood ILD syndrome (chILD) has also been introduced.

DLD is rarely observed in childhood. The prevalence varies, however, depending on the case definitions and ascertainment methods that have been defined in given epidemiologic studies. A study from the United Kingdom and Ireland estimated a prevalence of 3.6 cases per 1,000,000 children, while a German study (with different inclusion criteria) reported 1.32 cases per 1,000,000 children £16 years of age (6,7). Frequency estimates will likely increase as broader definitions and diagnostic methods of DLD are used, which will also result in reducing the proportion of unclassified ILD. Furthermore, as noted in the latest European Respiratory Review, chILD/DPLD represents an underdeveloped area of pulmonary research (1). With increasing accessibility to genetic testing, including multi-gene panels or even whole-exome/genome sequencing, a growing number of genetic factors (especially novel genes) are being identified as the causes of some forms of DLD in children. Hence, we support the need for collaboration among pneumologists, radiologists, pathologists and geneticists in both developing comprehensive chILD-categorisation systems and updating the database of molecularly defined pulmonary disease entities (1).

The study aims to present syndromic inherited disorders that may manifest with DLD, delineate their symptomatology and, consequently, improve knowledge on their diagnostic procedures.

### Clinical and genetic characteristic of syndromic disorders with ILD

From a geneticist point of view, the most practical classification of any inherited condition distinguishes their isolated and syndromic forms. In the context of children's DLD, these may include disorders with isolated chILD and syndromic diseases in which chILD is one of the features. Genetic conditions are classified in both these categories. Disorders of growth and development of lungs or inherited disorders of surfactant production and metabolism are examples of isolated pulmonary involvement, while, a variety of inherited errors of metabolism (IEM), such as lysosomal storage disorders (LSDs) or Hermansky–Pudlak syndrome, should be categorised as syndromic disorders with pulmonary involvement (associated with DLD).

For the purpose of this study, we searched Online Mendelian Inheritance in Man (OMIM^®^), the online catalogue of human genes and genetic disorders, for the terms “interstitial lung/pulmonary disease”. As a result, 40 entities were found. Several of these are congenital multi-systemic forms that we further discuss below (listed in Table 1). Inborn errors of metabolism are not included herein. Physicians should, however, keep in mind this group of disorders, especially, Gaucher disease, lysinuric protein intolerance, cobalamin C deficiency or Niemann–Pick disease type B, which may manifest with ILD.

**Table 1:** Congenital multisystem disorders manifesting with DLD

**Disease (phenotype)**	**MIM number**	**Chromosomal location**	**Gene/locus**	**Inheritance**	**Pulmonary involvement**
COPA syndrome; autoimmune interstitial lung, joint, and kidney disease (AILJK)	#616414	1q23.2	*COPA*	Autosomal dominant	DLD, haemorrhage, lymphocytic interstitial infiltration, ground-glass opacities on X-ray (8) DAH, DPLD (100% cases) (9)
Hermansky–Pudlak syndrome (1, 2, 4)	#203300, #608233, #614073	10q24.2, 5q14.1, 22q12.1	*HPS1, AP3B1, HPS4*	Autosomal recessive	Restrictive lung disease, recurrent infections, pulmonary fibrosis has been described largely in affected individuals from northwestern Puerto Rico (10); typically manifesting in the early 30s
Chitayat syndrome (CHYTS)	#617180	19q13.2	*ERF*	Autosomal dominant	Respiratory distress at birth, bronchomalacia and (or) tracheomalacia, complicated by recurrent severe respiratory infections, obstructive pulmonary disease, ILD (11)
Dyskeratosis congenita (DKCA, DC), including Hoyeraal–Hreidarsson syndrome and Revesz syndrome	#613990, #613989, #127550, #305000	14q12, 5p15.33, 3q26.2, Xq28	*TINF2, TERT, TERC, DKC1*	Autosomal dominant (TINF2, TERT, TERC); Autosomal recessive (TERT); X-linked (DKC1)	Idiopathic pulmonary fibrosis (12) DC should be considered in young persons with idiopathic pulmonary fibrosis (13)
Brain–lung–thyroid syndrome (BLTS); choreoathetosis and congenital hypothyroidism with or without pulmonary dysfunction (CAHTP)	#610978	14q13.3	*NKX2-1*	Autosomal dominant	ILD, neonatal respiratory distress, pulmonary fibrosis (14)
Interstitial lung and liver disease (ILLD)	#615486	12q13.3	*MARS*	Autosomal recessive	ILD, lung fibrosis, pulmonary artery malformation (15); pulmonary alveolar proteinosis (16)
Neurodevelopmental disorder with brain, liver and lung abnormalities (NEDBLLA); Rajab syndrome	#618007	2q36.1	*FARSB*	Autosomal recessive *(Autosomalnie recesywne)*	ILD (usually starts at the upper lobes), cholesterol pneumonitis (17)
- (reported in one patient so far)	- (reported once)	2q36.2	*FARSA*	Autosomal recessive	ILD with cholesterol pneumonitis, growth delay, hypotonia, brain calcifications with cysts and liver dysfunction (18)
Aarskog–Scott syndrome (AAS)	#305400	Xp11.22	*FGD1*	X-linked recessive	ILD reported once (19)
Chromosome deletion syndrome, including 14q11-q22 region		14q11-q22	Contiguous gene deletion	Isolated cases	Heterogeneous phenotype, depending on the size, but, mostly, on the genes located within the deleted region

COPA = the alpha subunit of the coatomer protein complex-I (viz. COPI); DLD = diffuse lung disease; DAH = diffuse alveolar haemorrhage; DPLD = diffuse parenchymal lung disease; ILD = interstitial lung disease

### COPA syndrome –

COPA syndrome is named after the causative gene, encoding the alpha subunit of the coatomer protein complex-I (i.e. COPI), which is required for retrograde protein trafficking from the Golgi apparatus to the endoplasmic reticulum (8). It is a newly recognised disease presenting in childhood. Familial occurrence is, however, mostly described. The clinical features vary, including even asymptomatic cases. Suggestive clinical characteristics include the following:

Main features: ILD, inflammatory arthritis and immune complex-mediated renal disease, accompanied by high-titer autoantibodies (8);
Rare characteristics: Neuromyelitis optica, extrapulmonary cysts (in liver and kidney), malignancies (i.e. carcinoid tumour, renal cell carcinoma) (20);
Additional laboratory findings: Anti-neutrophil cytoplasmic antibody, and(or) antinuclear antibody, as well as rheumatoid factor positivity (in 71% cases of Tsui et al. (9));
Miscellaneous: Incomplete penetrance in familial cases, and variable expression; symptoms appear before the age of 12 years, even at 1 year of age (9,20)

### Hermansky–Pudlak syndrome

HPS is a genetically heterogeneous disorder, in which pathogenic variants in 10 known genes result in dysfunction of four protein complexes that are involved in intracellular vesicle formation and trafficking: AP-3 (*AP3B1* and *AP3D1*genes), BLOC-1 (*BLOC1S3*, *BLOC1S6*, and *DTNBP1* genes), BLOC-2 (*HPS3, HPS5*, and *HPS6* genes) and BLOC-3 (*HPS1* and *HPS4* genes) (21). Depending on molecular pathology, the manifestation may differ, although most characteristics are as follows:

Main clinical features: Oculocutaneous albinism (nystagmus noted at birth, skin colour at least a shade lighter than that of other family members (22)) and bleeding diathesis;
Rare characteristics: Depend on the HPS subtype and may include pulmonary complications (as mentioned in Table 1), colitis (resembling Crohn's disease (23)), neutropenia, cardiomyopathy and renal failure (24);
Additional laboratory findings: Absence of delta granules (dense bodies) on whole-mount electron microscopy of platelets (24) and prolonged bleeding time;
Miscellaneous: Clinical and genetic heterogeneity, including increased susceptibility to infections in AP-3-deficient patients, and lethal pulmonary fibrosis in individuals with BLOC-3 deficiency (*HPS1, HPS4* variants).

### Chitayat syndrome

Chitayat et al. (25) first described it in 1993, in a 5.5-month-old boy with diffuse bronchomalacia, facial dysmorphism and digital anomalies. The follow-up at 21 years of age revealed obstructive pulmonary disease with ratio of forced expiratory volume in 1 second to forced vital capacity (FEV1/FVC ratio) of 52%, no response to bronchodilators and mild exertional dyspnoea (11). In general, the major traits reported to date for this condition are facial dysmorphism, hyperphalangism and respiratory complications in the newborn period. In more detail, these are as follows:

Main clinical features: Recognisable facial dysmorphism, including square face shape, hypertelorism, prominent eyes, depressed nasal bridge (in infancy) with upturned nasal tip, short columella and full lips, congenital anomaly of limbs such as brachydactyly and short index fingers with ulnar deviation, accessory phalanges on radial aspect of index fingers (on X-ray), bilateral hallux valgus and pectus excavatum;
Rare characteristics: Language and (or) motor delay (11); Miscellaneous: Polyhydramnios in the prenatal period.

### Dyskeratosis congenita

It is one of the disorders of telomeres, characterised by short telomeres for individuals’ age. To date, in approximately 70% of patients who meet the clinical diagnostic criteria for DC, pathogenic variants in *ACD, CTC1, DKC1, NHP2, NOP10, PARN, RTEL1, TERC, TERT, TINF2* and *WRAP53* genes have been identified (13). Since the first authors who described DC - FZ MFE and HN (26), its phenotype, has been delineated and includes the following:

Main clinical features: Clinical triad including dysplastic nails, lacy reticular pigmentation and atrophy of the skin, especially in the neck and upper chest region; and oral leucoplakia; moreover, any two or more of the physical abnormalities delineated by Vulliamy et al. (27) are required to establish the clinical diagnosis;
Rare characteristics: Increasing risk of progressive bone marrow failure (BMF) with age, myelodysplastic syndrome (MDS) or acute myelogenous leukaemia (AML), as well as solid tumours (typically squamous cell carcinoma of head and neck or anogenital adenocarcinoma) (13);
Additional laboratory findings: Shortened telomeres noted in automated multi-colour flow cytometry fluorescence in situ hybridisation (flow-FISH) on white blood cells subsets (28);
Miscellaneous: Wide phenotypic spectrum and age of onset, with genetic involvement and association with progressive telomere shortening; significant developmental delay in two DC variants, with additional findings that include cerebellar hypoplasia (Hoyeraal – Hreidarsson syndrome) and bilateral exudative retinopathy and intracranial calcifications (Revesz syndrome) (29).

### Brain–lung–thyroid syndrome

It belongs to the group of *NKX2-1*-related disorders that may manifest as abnormalities in a single organ system (as delineated below) or as any combination of brain, thyroid and lung involvement (thus referred to as “Brain–lung–thyroid syndrome” [BLTS]). In detail, it is characterised by the following:

Main clinical features: The triad of hypothyroidism, benign chorea and neonatal respiratory distress/ILD; isolated pulmonary symptoms in individuals with *NKX2-1* mutations are possible;
Rare characteristics: Muscular hypotonia in the neonatal period and early childhood, psychomotor delay (14), benign hereditary chorea (BHC) – which is the allelic disorder of LTB syndrome; giving the varied manifestations of *NKX2-1* mutations; it is now suggested that these disorders be referred to as NKX2-1-related disorders (30);
Additional laboratory findings: Any additional or atypical clinical features of classical BLTS may suggest deletions in 14q and should prompt expanded molecular diagnostics (31);
Miscellaneous: The *NKX2-1* gene was previously known as *TITF-1.*

### Interstitial lung and liver disease

The disorder is characterised by the onset of respiratory insufficiency and progressive liver disease in infancy or early childhood. Apart from the main features listed below, kidney stones, acetabular dysplasia, prolonged fever and extreme leucocytosis were noted.

Main clinical features: Respiratory insufficiency, liver dysfunction (as abnormal liver enzymes and hepatomegaly, cholestasis and cirrhosis), failure to thrive and developmental delay;
Rare characteristics: Aminoaciduria, lactic acidosis, hypoproteinaemia and hyperammonaemia;
Additional laboratory findings: Anaemia, thrombocytosis; examination of lung lavage is consistent with pulmonary alveolar proteinosis (16);
Miscellaneous: Monoallelic mutations in the *MARS* gene have been identified in autosomal dominant late-onset Charcot-Marie-Tooth disease, axonal type, 2U [MIM 616280] (32,33).

### Neurodevelopmental disorder with brain, liver and lung abnormalities

A link between neurodevelopmental disorders encompassing brain, liver and lung abnormalities (NEDBLLA) and compound heterozygous mutation in the *FARSB* gene was noted for the first time by Antonellis et al. (34). Soon after, another publication (35) confirmed the phenotypes consisting of short stature, elevated liver enzymes or liver cirrhosis, cerebral and basal ganglia calcification. These are not, however, specific features. Thus, to establish the diagnosis, molecular diagnostics is essential. In the genotype–phenotype correlations, it is also worth to look and analyse other characteristics, as follows:

Rare characteristics: Hypotonia, intracranial aneurysms, renal disease and intestinal malrotation (35);
Additional laboratory findings: Hypoalbuminaemia;
Miscellaneous: Reduction in the FARSB and FARSA proteins in western blot analysis, which may result from a reduction in phenylalanyl-tRNA synthetase activity.
Just recently, in patients with similar features (including ILD, hypotonia, growth delay and involvement of brain and liver), pathogenic variants were reported in the *FARSA* gene (18). Notably, similar to the FARSB disease, cholesterol accumulation and cholesterol clefts were found on lung biopsy.

### Aarskog-Scott syndrome (AAS)

The disease is also known as a faciodigitogenital syndrome or faciogenital dysplasia. Its estimated prevalence in the population is approximately 1 in 25,000, but the majority of patients have only clinical diagnosis with no subsequent molecular testing of the *FGD1* gene or with a negative result on tests. It is probably attributable to the clinical heterogeneity of AAS and(or) the fact that the clinical features overlap with those of other disorders, especially Noonan and Robinow syndromes (36).

Main clinical features: Short stature, facial dysmorphism (widow's peak, hypertelorism, ptosis, downslanting palpebral fissures and broad nasal bridge), genital malformation (shawl/bifid scrotum and cryptorchidism) and skeletal anomalies (brachydactyly, skin syndactyly and pectus excavatum) (37);
Comments: To the best of my knowledge, ILD in an individual with Aarskog syndrome has been reported once, by Escobar and Weaver (19). The patient had no molecular diagnosis (the *FGD1* gene has not been linked to the disease yet). More importantly, however, his facial dysmorphic features presented in the cited paper are not consistent with recognisable anomalies characteristic for the syndrome; hence, we question this identification.

## Conclusions

Aetiology of childhood DLD is varied and also includes inherited conditions. For their clinical classification, we propose to use isolated and syndromic entities, which allow distinguishing the conditions based on clinical presentation and depending on the underlying molecular pathology. In every child diagnosed with ILD, a detailed physical evaluation is necessary to decide whether a further genetic test is needed and to order a proper one (monogenic, multi-gene clinical sequencing or comprehensive genetic analyses).

Such a simple blood analysis can lead to the identification of the genetic variants consistent with the diagnosis, thus avoiding the need for further, even invasive, procedures during patients’ evaluations. Genetic testing is suggested especially for infants presenting with acute respiratory failure of unexplained aetiology and(or) in older children with chronic presentation and positive family history of DLD or complex syndromology (as described herein). Finally, when molecular pathology has already been established, clinical verification to prove genotype–phenotype relation and genetic counselling to the family should be offered.
